# “Seminal testosterone”, rising viewpoint of local spermatogenesis in nonobstructive azoospermia: One center long-term bidirectional cohort study

**DOI:** 10.3389/fendo.2022.992556

**Published:** 2022-12-08

**Authors:** Huang Liu, Houbin Zheng, Yuehua Li, Yuqian Tang, Hongbo Peng, Qianyi Li, Jiaming Zhuang, Yingyi Zhou, Yu Zhou, Xiang’an Tu, Xinzong Zhang

**Affiliations:** ^1^ Department of Andrology, National Health Commission (NHC) Key Laboratory of Male Reproduction and Genetics, Guangdong Provincial Reproductive Science Institute (Guangdong Provincial Fertility Hospital), Human Sperm Bank of Guangdong Province, Guangzhou, China; ^2^ Department of Clinical Laboratory, National Health Commission (NHC) Key Laboratory of Male Reproduction and Genetics, Guangdong Provincial Reproductive Science Institute (Guangdong Provincial Fertility Hospital), Human Sperm Bank of Guangdong Province, Guangzhou, China; ^3^ Department of Urology, The First Affiliated Hospital of Sun Yat-sen University, Guangzhou, China

**Keywords:** local spermatogenesis, reproductive hormone, seminal testosterone, bidirectional cohort study, non-obstructive azoospermia (NOA)

## Abstract

**Objective:**

Reproductive hormones are a traditional good method to evaluate spermatogenesis but might not accurately represent local spermatogenesis. To find a more accurate method, seminal reproductive hormones were studied.

**Methods:**

A bidirectional cohort study was performed. A total of 126 infertile men from 2018 to 2019 were retrospectively analyzed. They were divided into nonobstructive azoospermia (NOA), oligozoospermia (OLZ) and normal (NOR) groups. A prospective study was conducted on patients in the NOA and OLZ groups for 2 years. Microscopic testicular sperm extraction was performed for NOA patients, who were divided into a focal spermatogenesis group (FS) and an idiopathic azoospermia group (IA). Drug treatment was for OLZ patients, who were divided into a valid group (VA) and an invalid group (IN). The differences in sperm parameters and reproductive hormones were compared. ANOSIM analysis was used between and within groups. Pearson correlation analysis, CO inertia analysis and Proctor’s analysis were for relationships. ROC curve for the specificity and sensitivity. Time series analysis was for the trends between hormones and time.

**Results:**

The b-FSH, b-LH, s-T and ΔT in the NOA group were significantly higher than those in the OLZ and NOR groups. However, the s-FSH, s-E_2_, s-P, ΔFSH, ΔLH, ΔP and ΔE_2_ were lower. Thirty-one NOA patients underwent MTSE, of whom 12 had sperm (FS) and 19 had no sperm (IA). The s-FSH and s-E_2_ of the FS group were higher than those of the IA group. Twenty-six OLZ patients completed 30 days of treatment, of which 11 had an improved sperm count (VA) and 15 had no (IN). The ΔT of the VA group was higher than that of the IN group. After follow-up for 2 years, 18 patients’ results showed that b-FSH, b-LH and s-T were different over time, with delays of 19, 3 and -19 days. SC is closely related to pH, s-FSH, s-LH, s-E_2_, s-P, s-T, b-FSH, b-LH, ΔFSH, ΔLH, ΔP, ΔE_2_ and ΔT. There were complex common trends and relationships between different kinds of hormones. s-FSH, s-LH, s-E_2_, s-P, s-T, b-FSH and b-LH were useful to judge spermatogenesis, of which s-T, b-FSH and b-LH were more sensitive. If s-T, b-FSH and b-LH reached 64.4, 9.4 and 4.7, respectively, their prediction performance was the strongest.

**Conclusion:**

Seminal testosterone is sensitive for judging local spermatogenesis in nonobstructive azoospermia patients, which may be the direction of local spermatogenesis in nonobstructive azoospermia.

**Clinical trial registration:**

http://www.chictr.org.cn/index.aspx, identifier ChiCTR2200060463.

## Introduction

According to the World Health Organization, human infertility has become a worldwide problem, and its incidence is approximately 15%, half of which is caused by male factors; among them, 70% have abnormal sperm parameters (1, 2). Improving spermatogenesis and the quality of sperm is important for male infertility. Traditional theory has proven that reproductive hormones can reflect the state of spermatogenesis, which includes follicle stimulating hormone (FSH), luteinizing hormone (LH), prolactin (PRL), estradiol (E_2_), progesterone (P), and testosterone (T). There are positive regulation and negative feedback regulation mechanisms between these hormones, and their effective circulation promotes spermatogenesis (3, 4).

However, with the development of microscopic testicular sperm extraction, an increasing amount of local spermatogenesis has been found in the testes of patients with nonobstructive azoospermia (NOA) (5). Studies have shown that the presence of local spermatogenesis seems to have little relationship with peripheral blood reproductive hormones, which might not accurately predict the existence of focal spermatogenesis or have a limited effect on predicting local spermatogenesis (6–8). A meta-analysis showed that hormone therapy only improved the focal spermatogenesis of normal gonadotropin men but not high gonadotropin patients (9). According to the current research, the specificity and sensitivity of reproductive hormones in peripheral blood to predict local spermatogenesis are still controversial (10–13). This has become the focus of current academic debate.

To find a more accurate prediction method, we conducted a bidirectional cohort study. First, we retrospectively analyzed the seminal and blood reproductive hormones of males of childbearing age. Second, we observed the change trend of seminal and blood hormones and time of these men at different stages of spermatogenesis to find the rising viewpoint of local spermatogenesis in nonobstructive azoospermia and provide a reference to accurately predict spermatogenesis.

## Materials and methods

### Patient setting

The data of 126 infertile patients in the male outpatient department of the NHC Key Laboratory of Male Reproduction and Genetics, Guangdong Provincial Reproductive Science Institute (Guangdong Provincial Fertility Hospital), Human Sperm Bank of Guangdong Province, from January 2018 to December 2019 were randomly selected for retrospective cohort analysis. According to the sperm counts, they were divided into three groups: the nonobstructive azoospermia group (NOA group), oligozoospermia group (OLZ group) and normal group (NOR group). The age of the patients was between 21 and 54 years, with an average age of 33.2 ± 6.1 years. The height was between 156.0 and 183.0 cm, with an average age of 170.1 ± 6.4 cm. Their weight was between 52.4 and 89.9 kg, with an average age of 69.8 ± 8.8 kg. The BMI was between 18.3 and 30.4 kg/m^2^, with an average age of 24.1 ± 2.8 kg/m^2^. The reproductive hormones in seminal plasma and blood in the different groups were compared. The relationship between sperm parameters and these reproductive hormones was analyzed.

A prospective cohort study was conducted on patients in the nonobstructive azoospermia group (NOA Group) and oligozoospermia group (OLZ Group) who were followed up for 2 years. Under the principle of informed consent and voluntary selection, the NOA Group patients underwent microscopic testicular sperm extraction. According to whether they obtained sperm during the operation, they were divided into a focal spermatogenesis group (FS group) and an idiopathic azoospermia group (IA group). Drug treatment was performed for the OLZ group. After the treatment, the patients were divided into the valid group (VA group) and the invalid group (IN group). The reproductive hormones in seminal plasma and blood were compared before and after treatment. The correlation between the changes in reproductive hormones and the presence of focal spermatogenesis was analyzed by a time series model.

### Inclusion criteria

Patients included in the study must meet the following requirements: (1) Have the ability of independent behaviors, be able to ejaculate *in vitro* to collect semen and be willing to accept blood examination. (2) Be able to cooperate with physical examination and complete follow-up. (3) The chromosome and azoospermia factor gene are normal. (4) The body and external genitalia development are normal.

### Exclusion criteria

The exclusion criteria were as follows: (1) Obstructive azoospermia. (2) Genital tract infection. (3) Varicocele. (4) Cryptorchidism. (5) All those who did not meet the inclusion criteria. (6) All those who do not accept follow-up and quit halfway. (7) Familial hereditary disease. (8) Retrograde ejaculation. (9) Contraindications to surgery. (10) Long-term use of drugs or exposure to radiation and smoking and drinking habits.

### Diagnostic criteria

According to the WHO Manual on Laboratory Testing of Human Semen (2) and the diagnostic guidelines and expert consensus on male infertility (14), all the patients were divided into three population groups who met the relevant standards:

#### Normal sperm population

The sperm concentration is greater than or equal to 15 million/mL or the total number of sperm ejaculated each time is greater than or equal to 39 million/ejaculation.

#### Oligospermia population

The sperm concentration is less than 15 million/mL or the total number of sperm ejaculated each time is less than 39 million/ejaculation.

#### Nonobstructive azoospermia population

No sperm were found after two consecutive ejaculated semen centrifugations. FSH or LH increased or decreased, the testicular volume was smaller than normal, and no sperm were found in testicular puncture biopsy. Eliminate the obstructive azoospermia.

### Semen analysis

Fresh semen samples extracted by masturbation within 2-7 days of abstinence were collected and placed in a 37°C water bath shock box to be fully liquefied. Then, computer-aided semen analysis (CASA, Spanish) was used for the analysis of semen samples. The standard detection procedure was performed according to the WHO Manual for Detection and Analysis of Human Semen (2). If there were no sperm under normal light microscopy, the samples were centrifuged at 3000 g/min, and the sediment was re-examined for sperm.

### Hormone detection

ELISA was used for hormone detection. The instruments included the following: (1) Human ELISA KIT (FSH:G20171110 WN, E2:G20171110KS, P:G201711TO, LH:G20171111EV, T:G20171110JJ, 96T, Shanghai Langton Biotechnology Co., LTD); Thermostatic water bath (HWS-5A, Shanghai Baidian Instrument Equipment Co., LTD); Constant temperature shaker (BSD-YX(F)3200, Shanghai Boxun Industrial Co., LTD); Multifunctional enzyme marker detector (MULTISKANMK3, Thermo Scientific, USA). (1) Dilute the standard, biotin antigen and avidin HRP. (2) Developers A and B and termination solution were added to the blank well for zero adjustment. (3) A total of 50 µl of diluted standard was added to the standard well, and then 50 µl of enzyme-labeled reagent was added. (4) A 50 µl sample was added to the sample hole. Then, 50 µl of biotin antigen working solution and avidin-hrp50 µl were added successively. After incubation at 37°C for 30 min, chromogenic agent was added to avoid light for color development for approximately 10 min, and 50 µl termination solution was added. (5) The absorbance (OD) of each well was measured in sequence at a wavelength of 450 nm.

### Microscopic testicular sperm extraction

**Figure 1:**
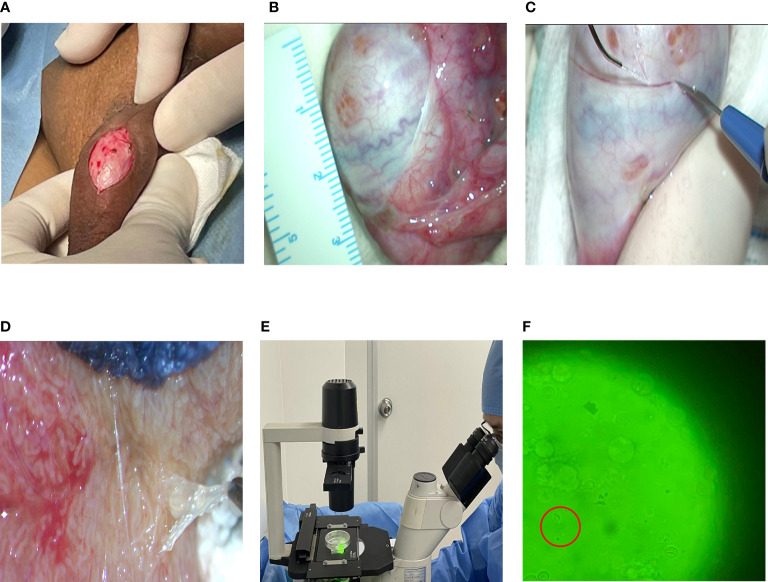
Flow chart of MTSE. **(A)** Skin incision. **(B)** Testicular measurement. **(C)** Incision of testicle. **(D)** Full seminiferous tubules. **(E)** Looking for sperm. **(F)** Sperm under a microscope (40x10).

All patients underwent microscopic testicular sperm extraction (M-TESE) with an Ultra high definition surgical microscope (Vario/S88, Zeiss, Germany) under the principle of informed consent. The steps were as follows: (1) Endotracheal intubation and general anesthesia were performed. (2) A longitudinal incision was made in the middle line of the scrotum with a length of approximately 1 cm (**Figure 1A**). The skin, flesh membrane and sheath membrane were cut successively, and the testis and epididymis were exposed. The development of the testis, epididymis and vas deferens was observed. (3) The axial diameter of the testis was measured (**Figure 1B**), the white membrane was cut along the equatorial plane of the testis, and the testicular endospermal tubules were exposed (**Figure 1C**). (4) An ultrahigh definition surgical microscope (Vario/S88, Zeiss, Germany) was used to search for fully developed spermatogenic tubules in the testis (**Figure 1D**), and these spermatogenic tubules were extracted and examined under an inverted microscope (Ti-U/B, Nikon, Japan) to determine whether there were sperm (**Figure 1E**). (5) If sperm is found, the operation is terminated (**Figure 1F**). If no sperm is found on one testicle, surgery continues on the other testicle. At the same time, testicular tissue was taken for pathological biopsy.

### Medical treatment

According to the guidelines of consensus on the treatment of oligoasthenospermia with integrated traditional Chinese and Western medicine (15) and referring to the wishes and needs of patients to improve sperm, the oligozoospermia group (OLZ Group) was treated by Chinese patent medicine. Traditional Chinese medicine (TCM) believes that oligozoospermia belongs to kidney deficiency and blood stasis. Therefore, the traditional Chinese patent medicine Shengjing capsule (Approval NO: Gyzz Z20027672, Specifications: 0.4×24#, Zunyi Liaoyuanhetang Pharmaceutical Co., Ltd, China) was used to increase the number of sperm under the principle of informed consent. The administration was 1.6 g, tid×30 days. In case of side effects such as dizziness or rash, the drug was stopped immediately and withdrawn from the treatment.

### Observation index

The indicator of total sperm count (SC) (million/ejaculation), age (year), height (cm), weight (kg), BMI (kg/cm2), days of abstinence (AT) (day), semen volume (SV) (mL), semen pH (pH), semen liquefaction time (LT) (min), blood reproductive hormones [Including follicle stimulating hormone (b-FSH) (mIU/mL), luteinizing hormone (b-LH) (mIU/mL), progesterone (b-P) (nmol/L), estradiol (b-E_2_) (ng/L) and testosterone (b-T) (nmol/L)], seminal plasma reproductive hormones[Including follicle stimulating hormone (s-FSH) (mIU/mL), luteinizing hormone (s-LH) (mIU/mL), progesterone (s-P) (nmol/L), estradiol (s-E_2_) (ng/L) and testosterone (s-T) (nmol/L)], the Δ value of the hormones(Δ value = seminal plasma index - serum index) [Including follicle stimulating hormone (ΔFSH) (mIU/mL), luteinizing hormone (ΔLH) (mIU/mL), progesterone (ΔP) (nmol/L), estradiol (ΔE_2_) (ng/L) and testosterone (ΔT) (nmol/L)] between the two fluids and the time for review (TR) [Including the first inspection time (TR1), the second recheck time (TR2) and the third recheck time (TR3)] were examined and recorded.

### Statistical methods

GraphPad Prism 8.0 (GraphPad Software, California, USA) was used for statistical analysis. Student’s *t* tests and *χ* ^2^ tests were used for comparisons in the groups. One-way ANOVA was used for pairwise comparisons. ANOSIM was used to assess differences between and within groups. Pearson correlation analysis, CO inertia analysis and Proctor’s analysis were used to analyze the correlations. ROC curves were used to judge the specificity and sensitivity. Time series analysis was used to determine the trend of the changes. *P<0.05* was defined as statistically significant.

## Results

### The follow-up results

**Figure 2:**
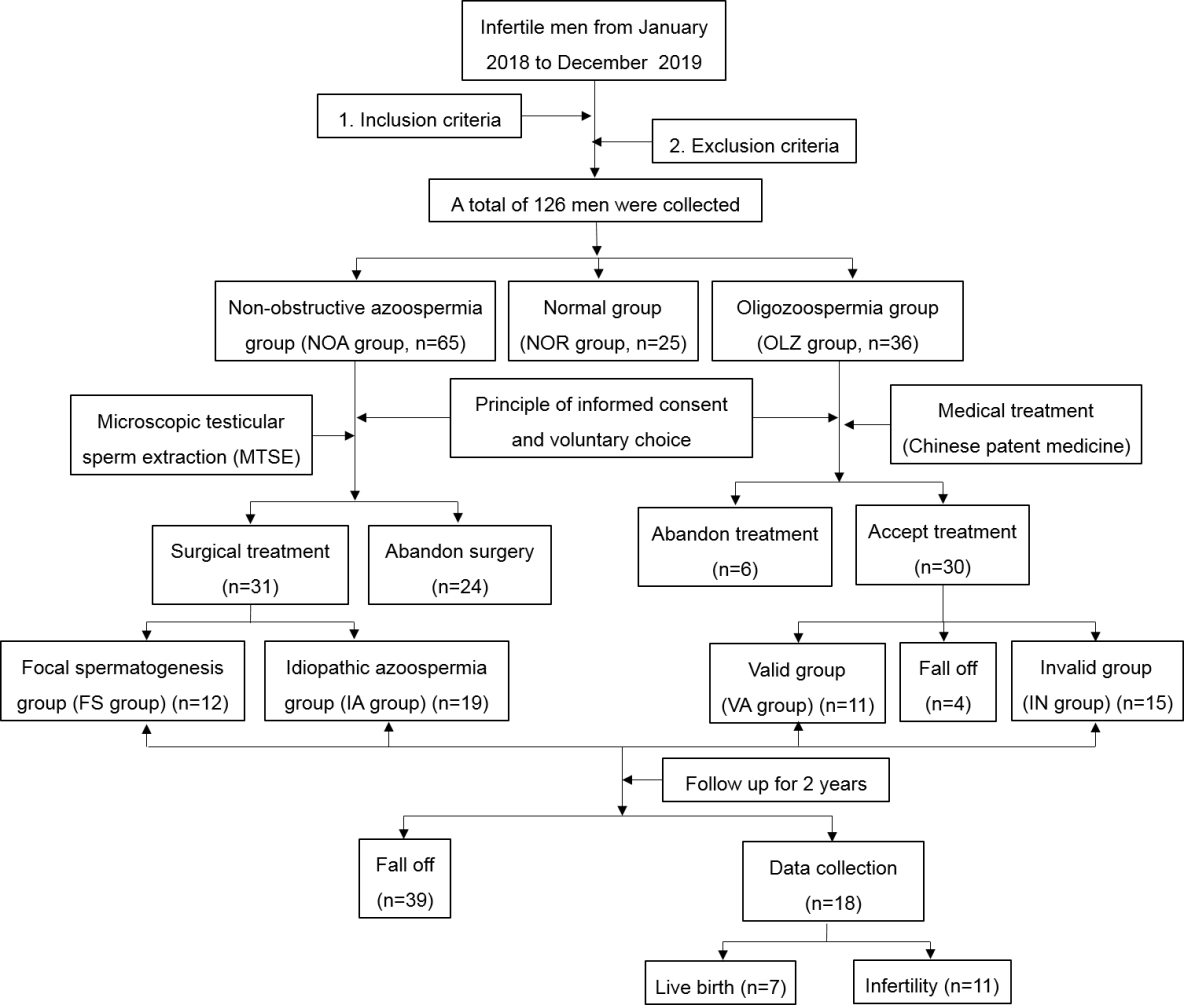
Flow chart of the study.

According to the inclusion criteria and exclusion criteria, data from a total of 126 men were collected from January 2018 to December 2019 for retrospective analysis. According to the total sperm counts, they were divided into a normal group (NOR group) with 25 patients, an oligospermia group (OLZ group) with 36 patients, and Nonobstructive azoospermia group (NOA group) with 65 patients. In the NOA group, 24 patients gave up the operation, and 31 patients received surgical treatment, among whom 12 patients obtained sperm through the surgery, but 19 did not. The sperm recovery rate (SRR) of MTSE was approximately 38.7%. In the OLZ group, 6 patients refused the treatment, and 30 patients accepted it. During the treatment, 4 patients withdrew from the observation due to cough, oral ulcer, constipation and other discomfort. After one month of treatment, the total sperm count of 11 patients increased, and 15 patients did not change or decrease. After the treatment, all the patients were followed-up for 2 years. There were 18 patients collected, among whom 7 were live births and 11 were infertile, with a live birth rate of 38.9%. (**Figure 2**).

### Comparison of the baseline data

One-way ANOVA showed that there was no difference in age, height, weight, BMI, AT, SV, pH, LT or other general baseline data among the NOA group, NOR group and OLZ group (all P>0.05) (**Table 1**).

**Table 1:** Comparison of the baseline data.

Indicators	NOR group	OLZ group	NOA group	F	P
Age (year)	31.4 ± 6.0	33.7 ± 7.8	33.7 ± 5.3	1.391	0.253
Height (cm)	171.7 ± 6.1	169.5 ± 6.5	169.7 ± 6.5	1.029	0.360
Weight (kg)	69.8 ± 8.5	67.9 ± 9.1	70.8 ± 8.8	1.221	0.298
BMI (kg/cm^2^)	23.7 ± 2.6	23.6 ± 2.7	24.5 ± 3.0	1.766	0.175
AT (day)	4.6 ± 1.6	4.7 ± 1.8	5.2 ± 2.5	0.744	0.447
SV (mL)	4.1 ± 1.2	3.6 ± 1.4	4.2 ± 1.4	2.284	0.106
pH	8.0 ± 0.3	8.0 ± 0.4	7.9 ± 0.4	2.100	0.127
LT (min)	26.5 ± 7.0	26.4 ± 6.0	28.5 ± 7.7	1.255	0.289

AT, days of abstinence; SV, semen volume; pH, semen pH; LT, semen liquefaction time. F, For F test.

### Comparison of hormones in different SC groups

**Figure 3:**
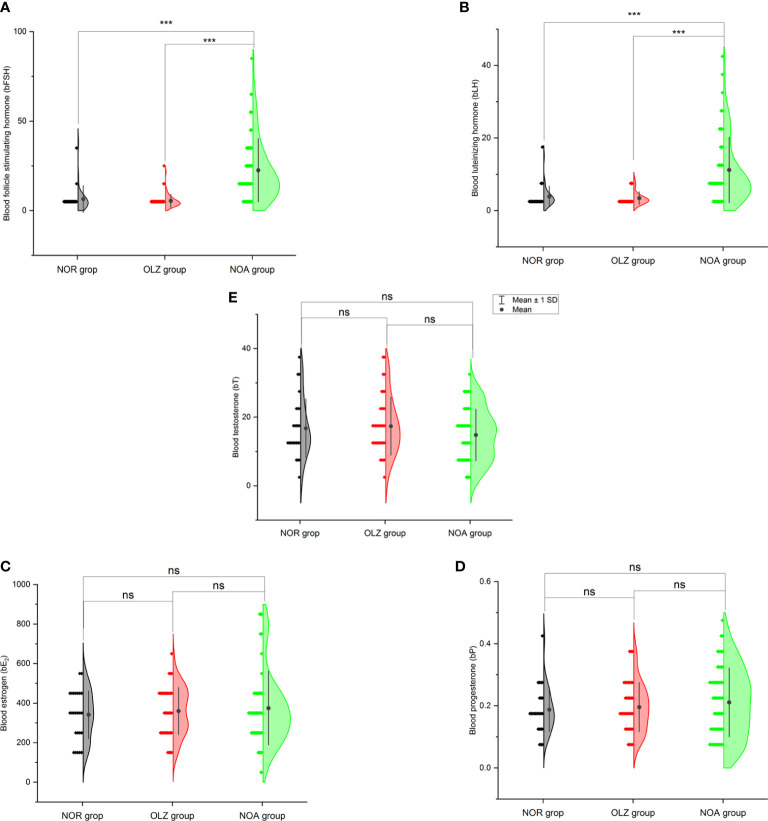
Comparison of blood hormones among the three groups. **(A)** Blood follicle stimulating hormone. **(B)** Blood luteinizing hormone. **(C)** Blood estradiol. **(D)** Blood progesterone. **(E)** Blood testosterone. ***: P<0.001; ns, no significance.

One-way ANOVA showed that b-FSH (22.5 ± 17.7) (**Figure 3A**) and b-LH (11.2 ± 9.0) (**Figure 3B**) in the NOA group were significantly higher than those in the OLZ group (5.5 ± 3.6; 3.4 ± 1.8) and NOR group (6.5 ± 7.5; 3.9 ± 2.8) (P<0.001), but there was no significant difference in b-E_2_ (**Figure 3C**), b-P (**Figure 3D**) and b-T (**Figure 3E**), and there was also no difference between the OLZ group and the NOR group.

**Figure 4:**
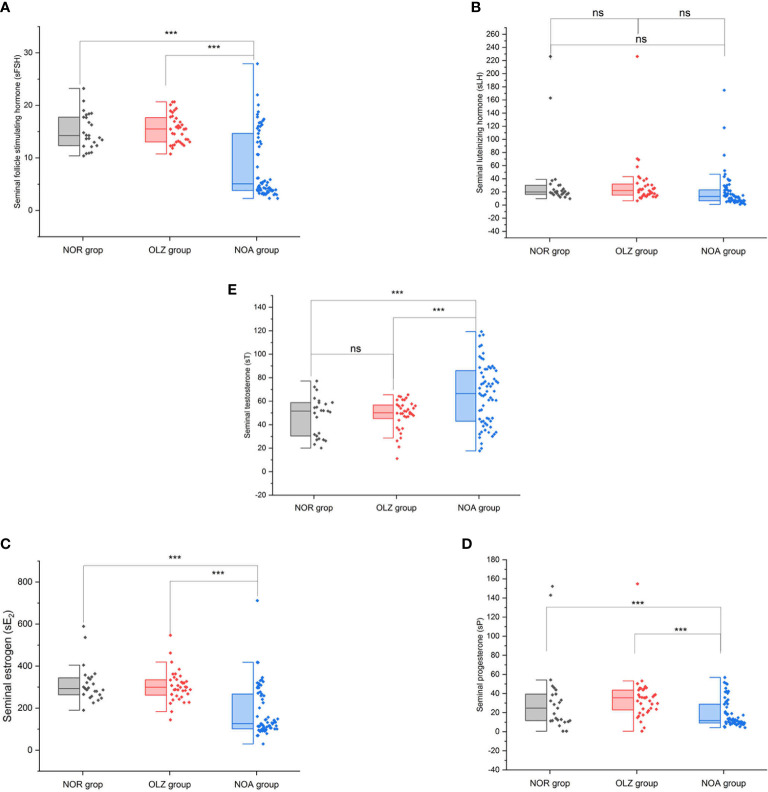
Comparison of seminal hormones among the three groups. **(A)** Seminal follicle stimulating hormone. **(B)** Seminal luteinizing hormone. **(C)** Seminal estradiol. **(D)** Seminal progesterone. **(E)** Seminal testosterone. ***: P<0.001; ns, no significance.

However, the results are very different in seminal plasma. The s-FSH (8.6 ± 6.4) (**Figure 4A**), s-E_2_ (181.3 ± 117.7) (**Figure 4C**) and s-P (18.9 ± 14.1) (**Figure 4D**) of the NOA group was significantly lower than those of the OLZ group (15.5 ± 2.7; 304.5 ± 74.5; 34.9 ± 24.3) and NOR group (15.1 ± 3.4; 315.2 ± 89.2; 33.4 ± 37.7), but the s-T (65.3 ± 25.8) was significantly higher than those of the OLZ group (48.5 ± 12.7) and NOR group (47.0 ± 16.7) (**Figure 4E**) (all P<0.001), and s-LH (20.4 ± 27.0) showed no difference (**Figure 4B**). However, there was no difference between the OLZ group and the NOR group.

**Figure 5:**
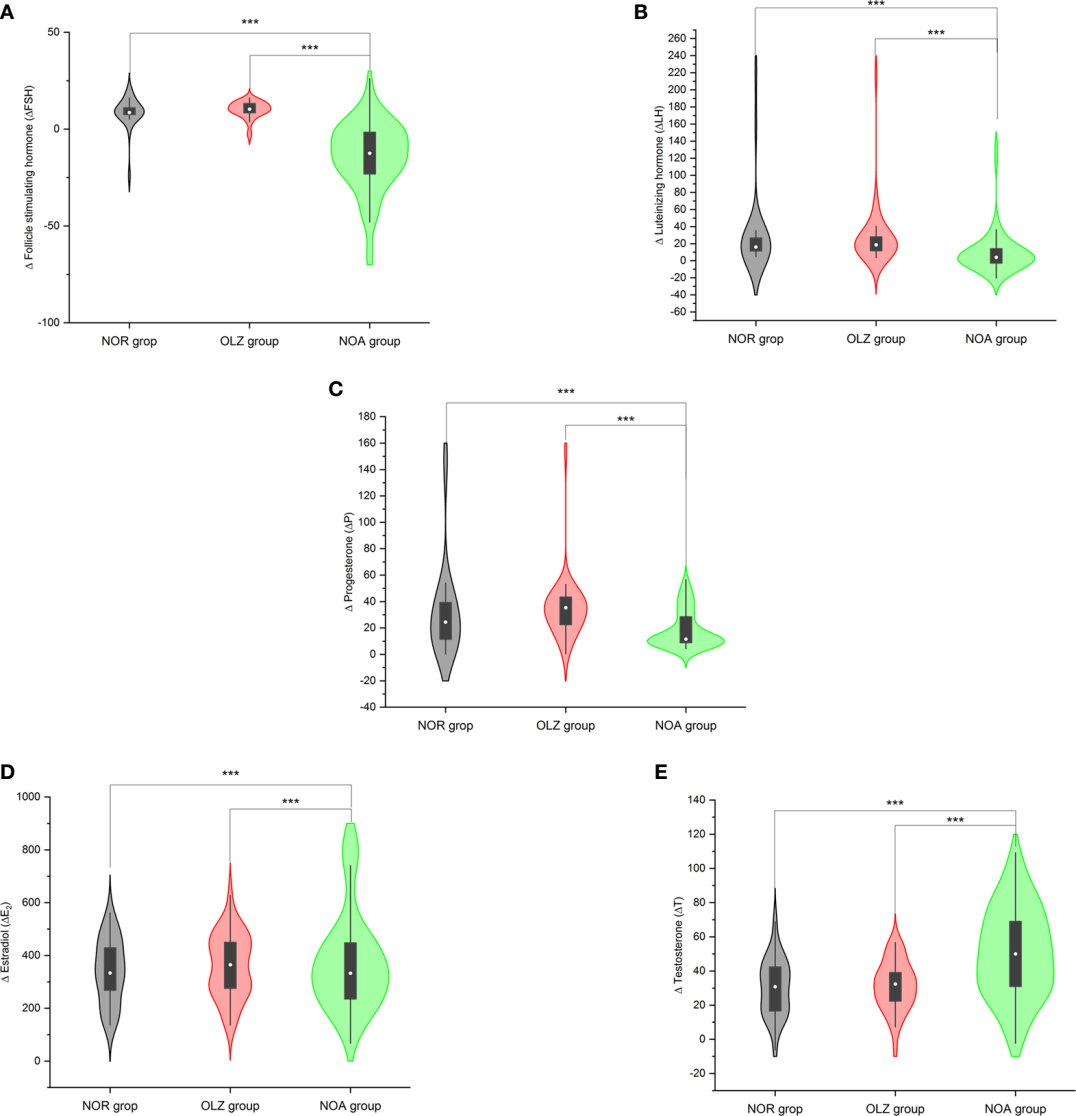
Comparison of Δ values of hormones among the three groups. **(A)** Δ follicle stimulating hormone. **(B)** Δ luteinizing hormone. **(C)** Δ estradiol. **(D)** Δ progesterone. **(E)** Δ testosterone. ***: P<0.001.

The Δ value of each hormone index in the NOA group was significantly different from that in the OLZ group and the NOR group, while there was no difference between the OLZ group and the NOR group. The ΔFSH (-13.9 ± 17.8), ΔLH (9.2 ± 25.0), ΔP (18.7 ± 14.1) and ΔE_2_ (-193.3 ± 226.9) in the NOA group were all smaller than those in the other two groups [(10.0 ± 4.3; 8.6 ± 8.1); (27.8 ± 35.9; 31.2 ± 49.7); (34.7 ± 24.3; 26.1 ± 24.5); (-55.5 ± 145.3; -26.1 ± 151.4)], while the ΔT (50.5 ± 26.4) was larger than those in the other two groups (31.1 ± 14.6; 30.3 ± 16.8) (**Figures 5A–E**).

### Comparison of hormones in the NOA group

**Figure 6:**
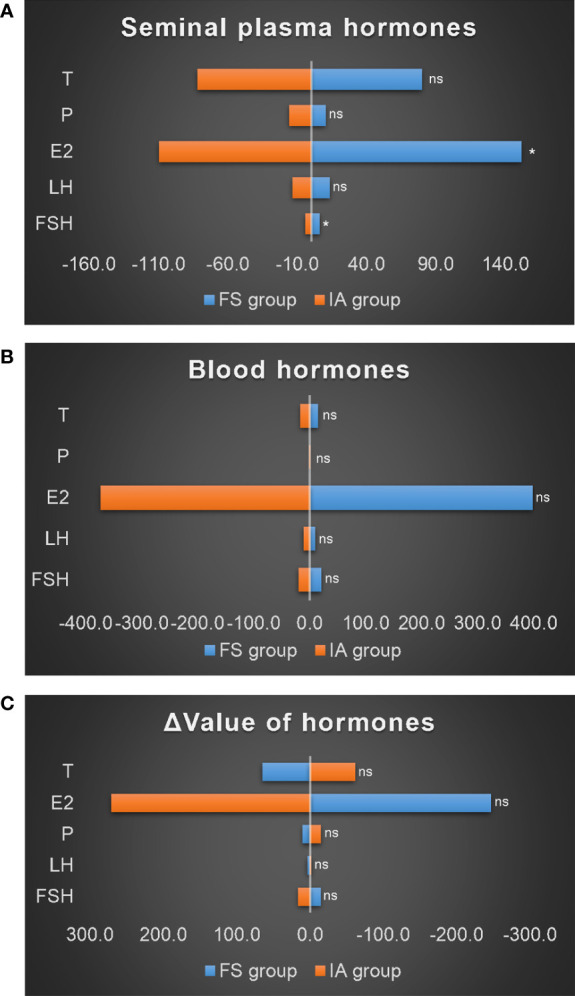
Comparison of hormones in the NOA group. **(A)** Seminal plasma hormones. **(B)** Blood hormones. **(C)** Δ value of hormones. *: P<0.05; ns, no significance.

Thirty-one patients in the NOA group underwent MTSE surgery, 12 patients had sperm (FS group) obtained from surgery, and 19 had no sperm (IA group). The s-FSH (6.0 ± 3.5) and s-E_2_ (151.6 ± 74.9) of the FS group were significantly higher than those of the IA group (3.9 ± 0.9; 102.9 ± 28.1) (P_1_ = 0.020; P_2_ = 0.015), but s-LH, s-P, and s-T showed no difference (**Figure 6A**). However, there was no difference in blood hormones between the two groups (**Figure 6B**) and no difference in the Δ value of hormones between the two groups (**Figure 6C**).

### Comparison of hormones in the OLZ group

**Figure 7:**
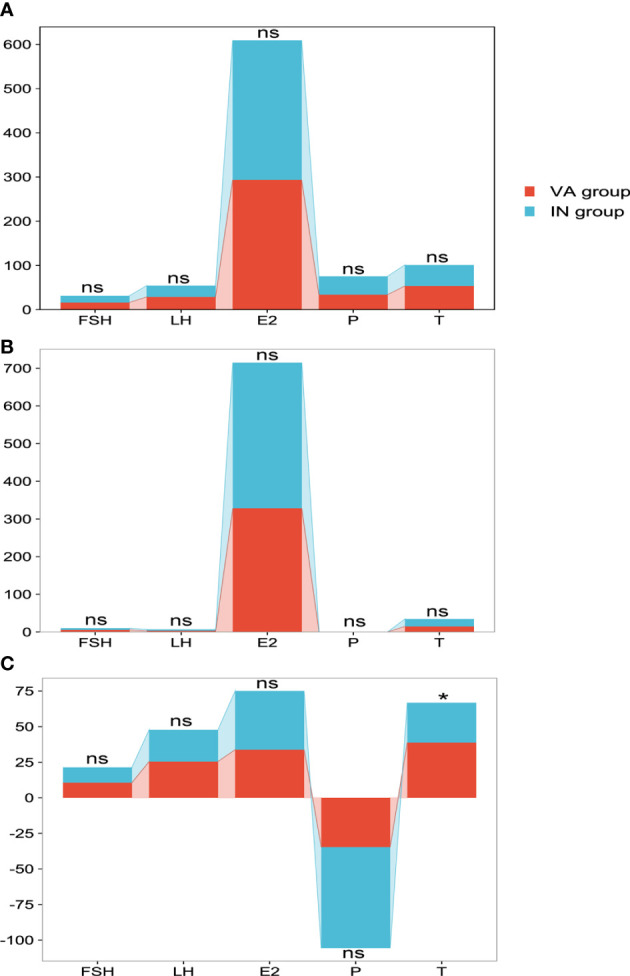
Comparison of hormones in the OLZ group. **(A)** Seminal plasma hormones. **(B)** Blood hormones. **(C)** Δ value of hormones. *: P<0.05; ns, no significance.

There were 26 patients in the OLZ group who received and completed the 30 days of TCM treatment, of which 11 had an improved sperm count (VA group) and 15 had no (IN group). There was no difference in semen hormones between the VA group and the IN group (**Figure 7A**) and no difference in blood hormones (**Figure 7B**). However, the ΔT (38.8 ± 10.9) of the VA group was significantly higher than that of the IN group (28.0 ± 11.3) (P=0.022), but there was no difference in the other indexes (**Figure 7C**).

### Changes in hormones in patients were followed up

**Figure 8:**
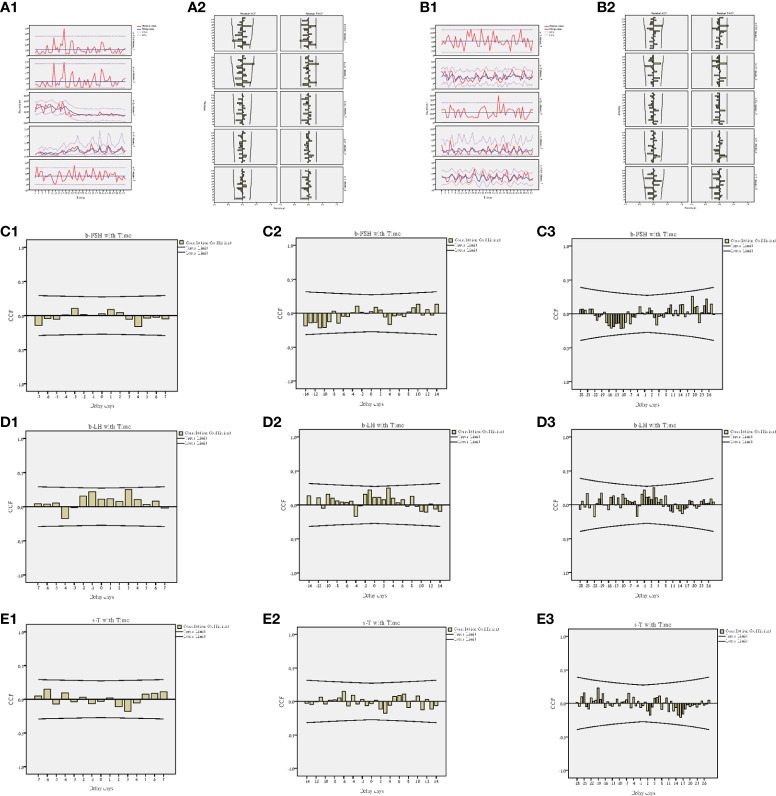
Time series model of hormones. **(A1)** Trend of blood hormones and time. **(A2)** ACF and PACF of blood hormones and time. **(B1)** Trend of seminal hormones and time. **(B2)** ACF and PACF of seminal hormones and time. **(C1)** Interaction between b-FSH and time at 7-day intervals. **(C2)** Interaction between b-FSH and time at 14-day intervals. **(C3)** Interaction between b-FSH and time at 28-day intervals. **(D1)** Interaction between b-LH and time at 7-day intervals. **(D2)** Interaction between b-LH and time at 14-day intervals. **(D3)** Interaction between b-LH and time at 28-day intervals. **(E1)** Interaction between s-T and time at 7-day intervals. **(E2)** Interaction between s-T and time at 14-day intervals. **(E3)** Interaction between s-T and time at 28-day intervals.

During the 2-year follow-up, 18 patients’ semen and blood hormones were examined twice and terminated when the spouse developed pregnancy or the patient gave up. Combined with the first hormone test before the treatment, 54 hormone tests were performed in 18 patients, and the changes in the hormones were analyzed based on a time series model. The results showed that the changes in FSH (b-FSH) and LH (b-LH) in the blood were significantly different over time (**Figure 8A1**), while the changes of T in the seminal plasma (s-T) were significantly different (**Figure 8B1**), but other indicators were not significant (**Table 2**).

**Table 2:** Time series model analysis.

Model	Number of predictive variables	Model fitting statistics					Ljung-Box Q (18)			Number of outliers
		Steady R^2^	R^2^	RMSE	MaxAPE	Normalized BIC	Statistic	DF	Sig.
**b-FSH-Model_1**	**0**	0.000	0.000	8.897	334.961	4.445	34.262	18.000	0.012*	**0.000**
**b-LH-Model_2**	**0**	0.000	0.000	5.366	481.939	3.434	43.588	18.000	0.001**	**0.000**
**b-E_2_-Model_3**	**0**	0.296	0.487	110.699	768.207	9.487	15.019	17.000	0.594	**0.000**
**b-P-Model_4**	**0**	0.360	0.419	0.153	299.998	-3.527	11.613	17.000	0.823	**0.000**
**b-T-Model_5**	**0**	0.000	0.000	7.727	4701.794	4.163	15.287	18.000	0.642	**0.000**
**s-FSH-Model_1**	**0**	0.283	0.283	5.514	336.360	3.636	22.183	16.000	0.137	**0.000**
**s-LH-Model_2**	**0**	0.135	0.088	11.986	283.994	5.115	22.884	17.000	0.153	**0.000**
**s-E_2_-Model_3**	**0**	0.000	0.000	106.415	219.877	9.409	13.679	18.000	0.750	**0.000**
**s-P-Model_4**	**0**	0.144	0.144	13.333	2589.740	5.328	13.956	17.000	0.670	**0.000**
**s-T-Model_5**	**0**	0.000	0.000	24.849	254.412	6.500	31.791	18.000	0.023*	**0.000**

b-FSH, blood follicle stimulating hormone; b-LH, blood luteinizing hormone; b-P, blood progesterone; b-E_2_, blood estradiol; b-T, blood testosterone; s-FSH, seminal follicle stimulating hormone; s-LH, seminal luteinizing hormone; s-P, seminal progesterone; s-E_2_, seminal estradiol; s-T, seminal testosterone. R^2^, Square of correlation coefficient r; RMSE, Root Mean Square Error; MaxAPE, Maximum relative percentage error; BIC, BIC Bayesian Information Criterion; Ljung-Box Q(18), Ljung-Box Q-test for autocorrelation; DF, Degree of freedom; Sig., Significance. *: p<0.05; **: p<0.01.

The cross-correlation analysis of time and hormones was carried out at three different time intervals of 7 days, 14 days and 28 days. It was found that the hormone change value with the highest correlation between b-FSH and time occurred when the delay day was 19 days (CCF=0.258) (**Figure 8C3**); b-LH occurred when the delay day was 3 days (CCF=0.250) (**Figure 8D1**); s-T occurred when the delay day was -19 days (CCF=0.229) (**Figure 8E3**).

### Correlation of seminal and blood hormones

**Figure 9:**
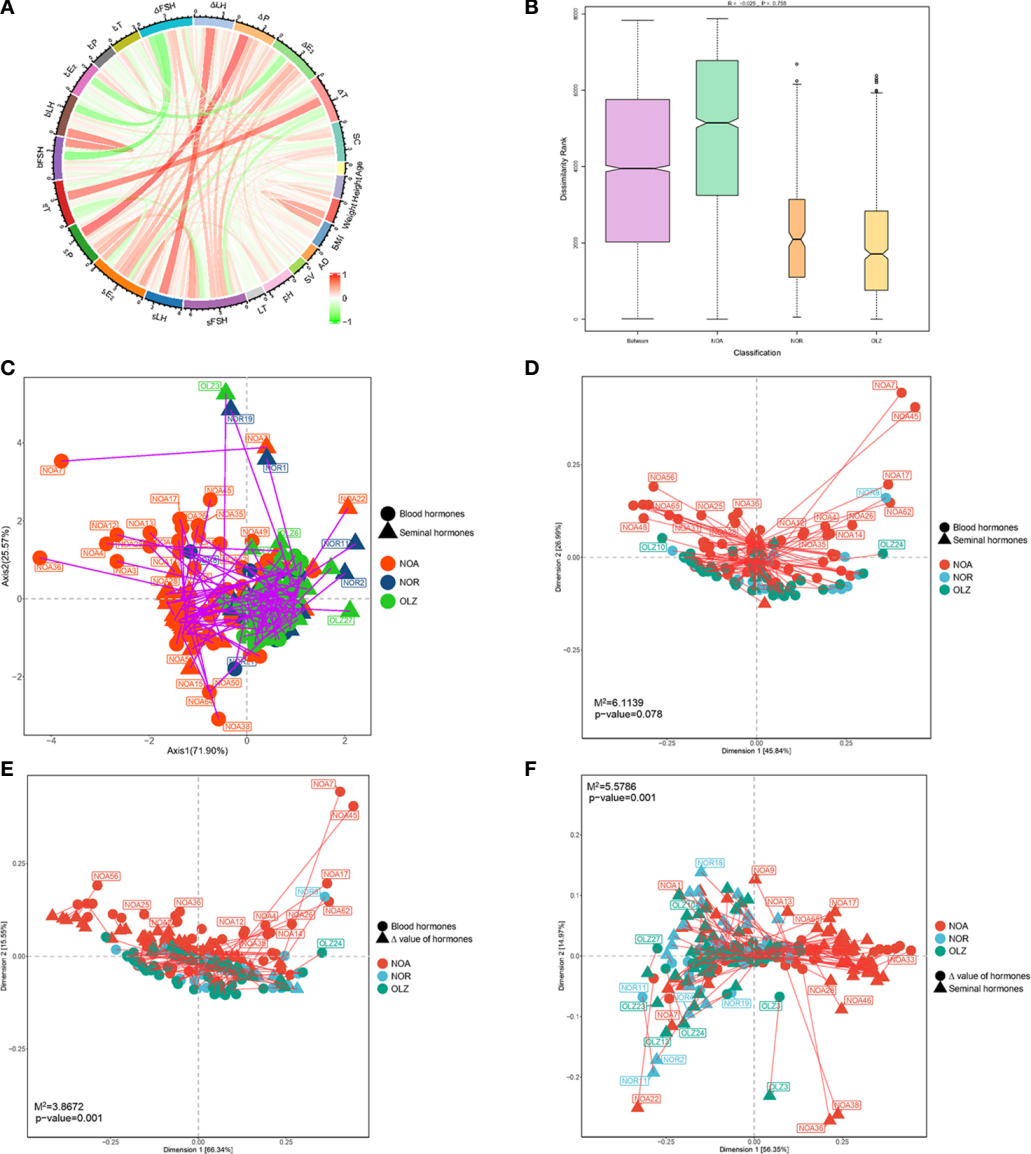
Correlation of seminal and blood hormones. **(A)** Relationship between indicators. **(B)** CO inertia analysis. **(C)** Procrustes analysis for seminal and blood hormones. **(D)** Procrustes analysis for seminal and blood hormones. **(E)** Procrustes analysis for blood hormones and Δ value of hormones. **(F)** Procrustes analysis for seminal hormones and Δ value of hormones.

Pearson analysis was used to analyze the relationship between total sperm count (SC) and age, height, weight, BMI, AT, SV, pH, LT, blood reproductive hormones [including b-FSH, b-LH, b-P, b-E_2_ and b-T], seminal plasma reproductive hormones [including s-FSH, s-LH, s-P, s-E_2_ and s-T] and the Δ value of the hormones [including ΔFSH, ΔLH, ΔP, ΔE_2_ and ΔT] between the two fluids in the first detection of all patients. The results showed the SC was closely related to pH (r=0.176,p=0.049), s-FSH (r=0.336,p<0.001), s-LH (r=0.178,p=0.046), s-E_2_ (r=0.381,p<0.001), s-P (r=0.257,p=0.004), s-T (r=-0.194,p=0.030), b-FSH (r=-0.271,p=0.002), b-LH (r=-0.253,p=0.004), ΔFSH (r=0.351,p<0.001), ΔLH (r=0.233,p=0.009), ΔP (r=0.257,p=0.004), ΔE_2_ (r=0.308,p<0.001) and ΔT (r=-0.209,p=0.019), but was without relationship with age, height, weight, BMI, AT, SV, LT, b-P, b-E_2_ and b-T (**Figure 9A**). CO inertia analysis showed that there was a close synergistic structure between seminal hormones and blood hormones, and there were complex common trends and relationships between the two (**Figure 9B**). ANOSIM (analysis of similarities) showed that there were differences in seminal and blood hormones between the NOA and OLZ and NOR groups (r=-0.025), but the overall differences were not significant (p=0.758) (**Figure 9C**). Procrustes analysis showed that although the overall differences were not significant (p=0.078) for seminal and blood hormones between the NOA and OLZ and NOR groups (**Figure 9D**), there were significant differences between semen hormones and the Δ value of hormones in these three groups (p=0.001) (**Figure 9E**), and there were also significant differences between blood hormones and the Δ value of hormones (p=0.001) (**Figure 9F**).

### Critical point of hormone indexes for regulating spermatogenesis

The ROC curve was used to judge the critical value of blood and seminal hormones regulating spermatogenesis in NOA patients. The results showed that seminal hormones (including s-FSH, s-LH, s-E_2_, s-P, s-T), blood FSH (b-FSH) and LH (b-LH) were significantly used to judge spermatogenesis (p<0.05) (**Table 3**).

**Table 3:** Area under ROC curve of hormone indexes.

Inspection result variables	Measure of area	Standard error	Progressive sig	95% Confidence interval	
				Lower limit	upper limit
s-FSH	0.207	0.042	0.000**	0.124	0.29
s-LH	0.296	0.047	0.000**	0.204	0.389
s-E_2_	0.178	0.039	0.000**	0.102	0.254
s-P	0.289	0.047	0.000**	0.196	0.381
s-T	0.702	0.049	0.000**	0.607	0.797
b-FSH	0.875	0.034	0.000**	0.809	0.94
b-LH	0.849	0.035	0.000**	0.781	0.917
b-E_2_	0.494	0.052	0.903	0.392	0.595
b-P	0.551	0.053	0.319	0.449	0.654
b-T	0.434	0.051	0.203	0.334	0.535

s-FSH, Seminal follicle stimulating hormone; s-LH, Seminal luteinizing hormone; s-E_2_, Seminal estradiol; s-P, Seminal progesterone; s-T, Seminal testosterone; b-FSH, Blood follicle stimulating hormone; b-LH, Blood luteinizing hormone; b-E_2_, Blood estradiol; b-P, Blood progesterone; b-T, Blood testosterone. **: p<0.01.

**Figure 10:**
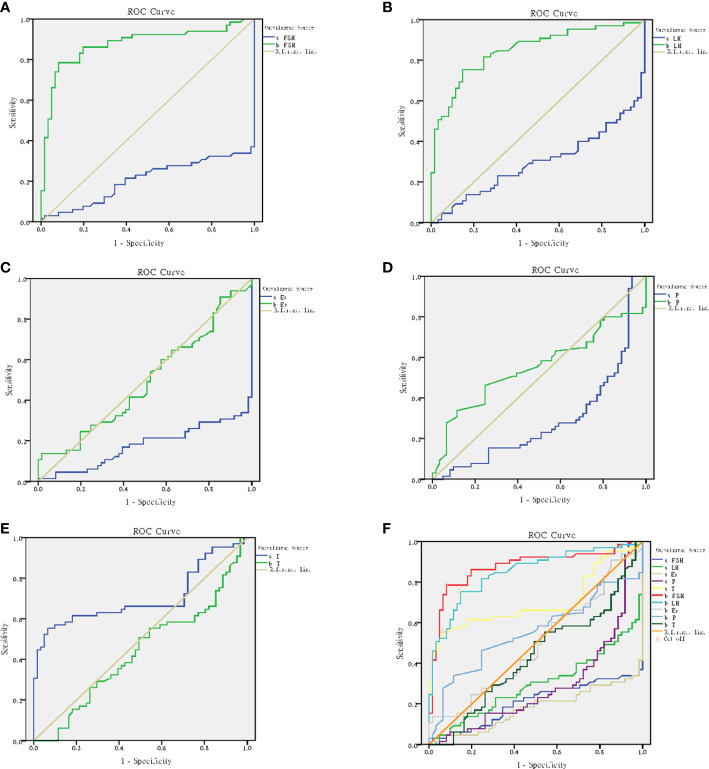
ROC curve analysis. **(A)** ROC curve for b-FSH and s-FSH. **(B)** ROC curve for b-LH and s-LH. **(C)** ROC curve for b-E_2_ and s-E_2_. **(D)** ROC curve for b-P and s-P. **(E)** ROC curve for b-T and s-T. **(F)** Cutoff for b-FSH, b-LH and s-T.

According to the results, s-FSH (AUC=0.207) (**Figure 10A**), s-LH (AUC=0.296) (**Figure 10B**), s-E_2_ (AUC=0.178) (**Figure 10C**), and s-P (AUC=0.289) (**Figure 10D**) had low diagnostic value in predicting spermatogenesis, while s-T (AUC=0.702) (**Figure 10E**), b-FSH (AUC=0.875) and b-LH (AUC=0.849) had high diagnostic value. When the optimum critical value of s-T was 64.4 (sensitivity=0.554, cutoff=0.488), b-FSH was 9.4 (sensitivity=0.785, cutoff=0.703), and b-LH was 4.7 (sensitivity=0.754, cutoff=0.606) (**Figure 10F**), they had the highest forecast accuracy.

## Discussion

A previous study showed that reproductive hormones played an important role in regulating spermatogenesis and in maintaining the number of spermatozoa (16, 17). The traditional view was that follicle stimulating hormone, luteinizing hormone and testosterone could promote spermatogenesis in seminiferous tubules by positive regulation and negative feedback regulation (18, 19). In recent years, studies have revealed that luteinizing hormone, follicle stimulating hormone and testosterone play a coordinated role in promoting spermatogenesis, and combined treatment with multiple hormones can achieve certain results. (20, 21). However, in patients with nonobstructive azoospermia, they do not seem to be so effective; the follicle stimulating hormone tends to remain high, but testosterone does not decrease. Moreover, there may be local spermatogenesis in their testicular group (6, 22). Some scholars even believe that the higher the follicle stimulating hormone is, the greater the possibility of local spermatogenesis (23).

In our study, we compared the reproductive hormones (contained follicle stimulating hormone, luteinizing hormone, progesterone, estradiol and testosterone) in the peripheral blood of the oligospermia group, normal sperm group and nonobstructive azoospermia group. We found that follicle stimulating hormone and luteinizing hormone levels in the nonobstructive azoospermia group were significantly higher than those in the other two groups, and there was no significant difference between the normal sperm and oligospermia groups. Our results are similar to those of some other studies (23–25). In these studies, researchers have suggested that the increase in FSH has an accurate predictive role for patients with NOA and that FSH can indicate the degree of damage to testicular spermatogenesis. In patients with NOA, FSH is negatively correlated with spermatogenesis, which is consistent with our research. However, according to their theory, FSH is linearly related to spermatogenesis, and there should be different gradient changes in populations with different degrees of spermatogenesis. However, our results do not support this assertion. Although we collected three populations with significant gradient differences in spermatogenesis, normal sperm population, oligozoospermia population and NOA population, we did not find that FSH or LH had gradient changes in the three populations, but there were only significant differences between the azoospermia population and sperm population. We believe that follicle stimulating hormone or luteinizing hormone may have a certain reference value for the existence of sperm, but there is not necessarily a specific linear relationship. From our results, we believe that follicle stimulating hormone or luteinizing hormone may not be continuous observation variables for the decline of sperm number, and the increase of follicle stimulating hormone and luteinizing hormone will not be coordinated with the declining trend of sperm number, they only judge and predict the presence or absence of sperm in semen, they should be a qualitative variable. This may be helpful to explain the viewpoint against the predictive function of reproductive hormones.

Paracrine of the testis is considered to be an important way to regulate spermatogenesis, and many cytokines, genes and proteins are involved in the paracrine pathway regulating spermatogenesis (26–29). In the testis, local follicle stimulating hormone, luteinizing hormone, progesterone, estradiol and progesterone are considered to be closely related to spermatogenesis (30, 31). Comparative medicine found that the local balance between androgen and estrogen was important for spermatogenesis (32). Therefore, these results suggested that there might be hormonal indicators more closely related to spermatogenesis in the local environment of spermatozoa, which was also one of the important tips to inspire our research.

We compared the differences between semen hormones and blood hormones. We found that the semen hormones of patients with nonobstructive azoospermia were significantly different from those of the oligospermia group and normal sperm group, and their differences were completely different from those of blood hormones. Zarezadeh et al. also found that both serum hormones and semen hormones have advantages and disadvantages in predicting spermatogenesis in patients with NOA (33). There may be some relationship between them, but there are significant differences. What is the relationship or difference between these serum hormones and semen hormones? At present, there are few studies. In this study, our analysis makes up for these gaps. We found that in semen, testosterone in patients with nonobstructive azoospermia significantly increased, while follicle stimulating hormone, progesterone and estradiol significantly decreased, but there was no difference in luteinizing hormone. These reproductive hormone indicators in semen are closely related to the number of sperm, which is of great value in judging spermatogenesis. In particular, seminal testosterone, blood follicle stimulating hormone and blood luteinizing hormone in plasma have high sensitivity and specificity for judging spermatogenesis. They also show periodic changes with time.

This first discovery surprised us. We preliminarily consider that there may be three reasons: First, seminal hormones came from the testicular microenvironment, which was more likely to cause a decline in sperm quality; when the seminal plasma hormone levels declined, the testosterone in seminal plasma would also be low, spermatogenesis could not obtain enough starting energy, so the spermatogenesis ability would decline.

Second, the source of serum hormones was the same as that of peripheral blood, which was more evenly distributed throughout the whole body. It was more accurate for the evaluation of hypothalamus pituitary function, which might be the reflection of the negative feedback of testis hypothalamus pituitary triggered by the decline of sperm quality. When sperm production decreased, follicle stimulating hormone and luteinizing hormone secreted into peripheral blood by the central nervous system increased, which showed that serum follicle stimulating hormone and luteinizing hormone increased. However, they did not directly act on testicular seminiferous tubules. Therefore, their changes did not match spermatogenesis. This explained why we could judge the decrease in spermatogenesis in the testis when we observed high follicle stimulating hormone or luteinizing hormone in the clinic, but we could not accurately predict the degree of spermatogenesis decrease, and we could not judge whether there was focal spermatogenesis. At this time, if we combined seminal plasma hormones, it might be more accurate to predict whether there was focal spermatogenesis in the testis. Therefore, high follicle stimulating hormone and luteinizing hormone in serum indicated decreased spermatogenesis, but high follicle stimulating hormone and luteinizing hormone in plasma indicated enhanced spermatogenesis. Serum hormones may be the result of spermatogenesis, but plasma hormones may be the cause.

Finally, although we did not detect follicle-stimulating hormone and luteinizing hormone receptors in testicular tissue, we detected testosterone, which is the product of follicle-stimulating hormone and luteinizing hormone acting on Sertoli cells and Leydig cells in testicular tissue, respectively (34–37). We found that the seminal testosterone of the azoospermia group was significantly higher than that of the spermatozoa group. This is a very interesting and very confusing phenomenon, and few researchers have noticed why this phenomenon occurs. This revealed that follicle stimulating hormone and testosterone might have a certain balance relationship; when this relationship was in balance, it could play the best role in promoting sperm production.

Some research found that the lower the testosterone/estradiol ratio in seminal plasma, the better the spermatogenic function of the testis, but there is no consensus boundary point of these indexes (38, 39). One animal experiment also found that blood luteinizing hormone must cooperate with follicle stimulating hormone to trigger a spermatogenic signal (40). These previous studies have shown that the various reproductive hormones regulating spermatogenesis must coordinate with each other to achieve the effect of changing spermatogenesis. The single index is often not comprehensive enough to evaluate spermatogenesis. However, this is not the case. Because of the existence of the blood-testis barrier, the microenvironment in the testis forms an independent system. As a result of the coordination of reproductive hormones in the testicular microenvironment, and with the change of time, the reproductive hormone in the seminal plasma should be able to reflect spermatogenesis. As we found, especially testosterone in seminal plasma, its change is periodic, and it can independently reflect the spermatogenesis state and be more comprehensive enough to evaluate spermatogenesis.

## Conclusion

However, we realized that our small sample size may lead to some bias in our observations, and we need to verify our findings with a large multicenter study in the future. Based on the above analysis, we believe that seminal testosterone is sensitive for judging local spermatogenesis in nonobstructive azoospermia patients, which may be due to the rising view of local spermatogenesis in nonobstructive azoospermia.

## Data availability statement

The original contributions presented in the study are included in the article/supplementary material. Further inquiries can be directed to the corresponding authors.

## Ethics statement

The studies involving human participants were reviewed and approved by the ethics committee of Guangdong reproductive research institute (Guangdong reproductive hospital) [Approval No.2017(16)]. The patients/participants provided their written informed consent to participate in this study.

## Author contributions

HL presided over the whole work, performed surgery, reviewed the literature, analyzed data and drafted the manuscript. HZ and YZ collected the patients and performed surgery. YL, YT and HP were used for hormone detection. QL, JZ and YYZ were for sperm finding, XZ directed the detection and XT directed the writing. All authors contributed to the article and approved the submitted version.
